# Chromosomally integrated human herpesvirus 6: questions and answers

**DOI:** 10.1002/rmv.715

**Published:** 2011-11-04

**Authors:** Philip E Pellett, Dharam V Ablashi, Peter F Ambros, Henri Agut, Mary T Caserta, Vincent Descamps, Louis Flamand, Agnès Gautheret-Dejean, Caroline B Hall, Rammurti T Kamble, Uwe Kuehl, Dirk Lassner, Irmeli Lautenschlager, Kristin S Loomis, Mario Luppi, Paolo Lusso, Peter G Medveczky, Jose G Montoya, Yasuko Mori, Masao Ogata, Joshua C Pritchett, Sylvie Rogez, Edward Seto, Katherine N Ward, Tetsushi Yoshikawa, Raymund R Razonable

**Affiliations:** 1 Department of Immunology and Microbiology, Wayne State University School of MedicineDetroit, MI, USA; 2 HHV-6 FoundationSanta Barbara, CA, USA; 3 Children's Cancer Research InstituteVienna, Austria; 4 Service of Virology, Groupe Hospitalier Pitié-SalpêtrièreParis, France; 5 Department of Pediatrics, University of Rochester School of MedicineRochester, NY, USA; 6 Department of Dermatology, Bichat Claude Bernard HospitalParis, France; 7 Rheumatology and Immunology Research Center, Université LavalQuebec, Canada; 8 Departments of Pediatrics and Medicine, University of Rochester School of Medicine and DentistryRochester, NY, USA; 9 Hematology & Oncology, Baylor College of MedicineHouston, TX, USA; 10 Cardiology and Pneumonology, Charite University BerlinBerlin, Germany; 11 Institute for Cardiac Diagnosis & TreatmentBerlin, Germany; 12 Department of Virology, HUSLAB & University of HelsinkiHelsinki, Finland; 13 University of Modena and Reggio EmiliaEmilia–Romagna, Italy; 14 National Institute of Allergy and Infectious DiseasesNIH, Bethesda, MD, USA; 15 Department of Molecular Medicine, University of South FloridaTampa, FL, USA; 16 Department of Infectious Disease, Stanford UniversityStanford, CA, USA; 17 Division of Clinical Virology, Kobe UniversityKobe, Hyōgo, Japan; 18 Hematology, Blood Transfusion Center, Oita UniversityOita, Japan; 19 Department of Virology, CHRU DupuytrenLimoges, France; 20 Department of Molecular Oncology, Moffitt Cancer Center & Research InstituteUSA; 21 Department of Infection, University College LondonLondon, UK; 22 Department of Pediatrics, Fujita Health UniversityToyoake, Aichi, Japan; 23 Division of Infectious Diseases, Mayo ClinicRochester, MN, USA

## Abstract

Chromosomally integrated human herpesvirus 6 (ciHHV-6) is a condition in which the complete HHV-6 genome is integrated into the host germ line genome and is vertically transmitted in a Mendelian manner. The condition is found in less than 1% of controls in the USA and UK, but has been found at a somewhat higher prevalence in transplant recipients and other patient populations in several small studies. HHV-6 levels in whole blood that exceed 5.5 log10 copies/ml are strongly suggestive of ciHHV-6. Monitoring DNA load in plasma and serum is unreliable, both for identifying and for monitoring subjects with ciHHV-6 due to cell lysis and release of cellular DNA. High HHV-6 DNA loads associated with ciHHV-6 can lead to erroneous diagnosis of active infection. Transplant recipients with ciHHV-6 may be at increased risk for bacterial infection and graft rejection. ciHHV-6 can be induced to a state of active viral replication *in vitro*. It is not known whether ciHHV-6 individuals are put at clinical risk by the use of drugs that have been associated with HHV-6 reactivation *in vivo* or *in vitro*. Nonetheless, we urge careful observation when use of such drugs is indicated in individuals known to have ciHHV-6. Little is known about whether individuals with ciHHV-6 develop immune tolerance for viral proteins. Further research is needed to determine the role of ciHHV-6 in disease. Copyright © 2011 John Wiley & Sons, Ltd.

## INTRODUCTION

This is a review of chromosomally integrated human herpesvirus 6 (ciHHV-6) and its potential clinical implications (Table 1), in the form of a series of questions and answers. The major points are summarized in Table 2. In many areas, available data are insufficient to support evidence-based guidance, leaving us with our opinions. We provide a list of research questions (Table 3) to motivate studies that will allow more extensive evidence-based guidance to be offered in several years. Table 4 lists drugs associated with HHV-6 reactivation; an expanded version is provided in Supplemental Table 1. Background information about human herpesvirus 6 (HHV-6) and ciHHV-6 is available elsewhere [1–6].

**Table 1:** Clinical scenarios that may be associated with ciHHV-6

Misdiagnosis of active HHV-6 infection in ciHHV-6 individuals presenting with unconnected illnesses.
Incidental positivity of CSF PCR for HHV-6 in ciHHV-6 patients with CSF pleocytosis resulting in erroneous diagnosis and unnecessary treatment.
Persistence of high levels of HHV-6 genomes (high HHV-6 DNA copy numbers).
Transmission of ciHHV-6 hematopoietic cells from donor to recipient following allogeneic HSCT.
Presence of high levels of HHV-6 DNA in the non-hematopoietic tissues but not in the hematopoietic tissues of a ciHHV-6 individual who received a non-ciHHV-6 HCST.
Transplantation of a solid organ from an individual with ciHHV-6 to a recipient without ciHHV-6.
Potential for ciHHV-6 reactivation in immunocompromised hosts.
Potential for ciHHV-6 reactivation in individuals treated with certain drugs.
Increased risk of bacterial infection in SOT recipients with ciHHV-6.
Uncertainty as to whether to treat ciHHV-6 patients who have symptoms associated with HHV-6 activity, such as CNS dysfunction.

ciHHV-6, chromosomally integrated human herpesvirus 6; HHV-6, human herpesvirus 6; HSCT, hematopoietic stem cell transplantation; SOT, solid organ transplantation.

**Table 2:** ciHHV-6: Key points

**What is ciHHV-6**? ciHHV-6 is a condition in which the complete HHV-6 genome is integrated into the host germline genome and is transmitted in a Mendelian manner.
**Why does it matter**? The high HHV-6 DNA load in patients with ciHHV-6 can lead to misdiagnosis due to the incorrect assumption that the patient is experiencing active HHV-6 infection for which antiviral treatment might be warranted. A hypothetical risk is that clinical or environmental exposure to certain drugs and chemicals may inadvertently activate the virus in patients with ciHHV-6.
**How can ciHHV-6 be diagnosed**? HHV-6 levels in whole blood that exceed 5.5 log_10_ copies/ml are strongly suggestive of ciHHV-6. This can be confirmed if the ratio of viral to human genomes is about 1:1.
**Should ciHHV6 individuals be treated for HHV-6**? It is unknown if individuals with ciHHV-6 have viral activity that would benefit from intervention. Antiviral therapy might be warranted in individuals with ciHHV-6 if they have clinical manifestations compatible with those typically associated with HHV-6 disease in the immunocompromised, and alternative concurrent etiologies have been excluded.

ciHHV-6, chromosomally integrated human herpesvirus 6; HHV-6, human herpesvirus 6.

**Table 3:** Important areas for further research related to ciHHV-6

**What is the prevalence of ciHHV-6 in populations of various geographic, cultural, and socioeconomic origins?**
**What are the consequences of ciHHV-6?**
Retrospective outcome analysis
Is ciHHV-6 over-represented in some diseases?
For example, children with neurological disorders, Hodgkin's lymphoma, GVHD
Prospective analysis: cohorts of individuals with ciHHV-6
Is ciHHV-6 associated with atypical development in children?
Is ciHHV-6 associated with peculiar phenotypes (physical or psychological)?
Do ciHHV-6 transplant recipients have a greater risk of GVHD or other adverse outcome?
Are grafts from ciHHV-6 donors more likely to be rejected or otherwise fail?
**Can ciHHV-6 be activated *in vivo* by exposure to common drugs or chemicals?**
Additional cell culture experiments are required to confirm the finding that ciHHV-6 can be activated *in vitro* by chemicals such as HDAC inhibitors and hydrocortisone.
Are individuals with ciHHV-6 at increased risk when they take drugs known to activate HHV-6 including common anti-seizure drugs valproic acid and carbamazepine?
**Can individuals with ciHHV-6 acquire HHV-6 horizontally?**
**Are there increased risks for blood transfusion, HSCT or SOT when the donor or recipient has ciHHV-6?**
**What are the risks of transplacental transmission of HHV-6 by ciHHV-6 mothers?**

ciHHV-6, chromosomally integrated human herpesvirus 6; HHV-6, human herpesvirus 6; GVHD, graft-versus-host disease, HSCT, hematopoietic stem cell transplantation; SOT, solid organ transplantation; HDAC, histone deacetylase.

**Table 4:** Drugs associated with reactivation or enhanced replication of HHV-6 [a]

Category	*In vitro*	*In vivo* [b]
**HDAC inhibitor**	Trichostatin A [*]	
	Sodium n-butyrate	
**Antibiotics**	Amoxicillin	
		TMP–SMX
		Minocycline
		Vancomycin
		Dapsone
**Immunosuppressant**	Hydrocortisone [*]	
**Anti-inflammatory**		Sulfasalazine
**Anesthetic**		Trichloroethylene
**Anti-arrhythmic**		Mexiletine
**Anticonvulsants**	Carbamazepine [c]	Carbamazepine [c]
Valproic Acid [c]
		Zonisamide
		Phenobarbitol
		Lamotrigine
		Phenytoin
**NSAIDs**		Ibuprofen
		Naproxen
**Other**		Allopurinol
	TPA (12-O-tetradecanoylphorbol-13-acetate) [7]	

a See Supplementary Table for additional information and references.

b *In vivo* associations have been in the context of DIHS, DRESS, TEN, SJS, and AHS.

c This drug has HDAC inhibitor properties.

* Also shown to activate chromosomally integrated virus.

HHV-6, human herpesvirus 6; HDAC, histone deacetylase; DIHS, drug-induced hypersensitivity syndrome; DRESS, drug rash with eosinophilia and systemic symptoms; SJS, Stevens–Johnson syndrome; AHS; anticonvulsant-induced hypersensitiviy syndrome; TEN, toxic epidermal necrolysis; NSAIDs, non-steroidal anti-inflammatory drugs.

### What is human herpesvirus 6?

Human herpesvirus 6 is the collective name for HHV-6A and HHV-6B, which are two closely related herpesviruses that have a combined seroprevalence of >90% in adults. HHV-6B is typically transmitted via saliva and primary infection usually occurs between 6 months and 2–3 years of age. Many primary HHV-6B infections are not associated with any specific clinical features, although the virus causes roseola infantum (exanthema subitum or sixth disease) in ~30% of children, presenting with high-grade fever followed by a characteristic rash that is sometimes accompanied by benign febrile convulsions, and rarely by status epilepticus. Little is known about primary HHV-6A infection and its disease associations. For the 99% of the population without ciHHV-6, most become infected with the virus in the first 2 years of life, with the virus establishing latency in a small fraction of mononuclear cells.

Disease associated with HHV-6A and HHV-6B reactivations can occur in immunocompromised hosts, and rarely in immunocompetent individuals. HHV-6B reactivation often occurs in immunocompetent individuals during drug induced hypersensitivity syndrome (DIHS) and the related drug rash with eosinophilia and systemic symptoms (DRESS). Symptoms and conditions that have been firmly associated with HHV-6B in immunocompromised patients include exanthematous rash, fever, seizures 2005, encephalopathy 2009, limbic encephalitis 2007 and amnesia [10,11], cognitive dysfunction 2011, lymphadenopathy 2004, colitis 14 15 16, and hepatitis [17,18]. Less well established, and in some cases controversial associations have been reported for Stevens–Johnson syndrome (SJS) 2010, renal failure [20,21], hemophagocytic syndrome [22,23], myocarditis 24 25 26, pneumonitis 27 28 29, hypogammaglobulinemia 2004, and arteriopathies [31,32]. HHV-6A reactivation has been found in subsets of patients with multiple sclerosis [33,34], HIV infection 35 36 37, encephalitis 2007, and syncytial giant-cell hepatitis 2008 (reviewed in [40,41]). The vast majority of low-level HHV-6 reactivations in immunocompromised patients are asymptomatic.

### What is chromosomally integrated human herpesvirus 6?

“Chromosomally integrated human herpesvirus 6” denotes the condition in which the complete HHV-6 genome is integrated into the telomere of a host cell chromosome 2006. Because the viral DNA is integrated into the germline genome, ciHHV-6 can be inherited in a Mendelian manner, with a 50% chance of being passed to a child. In addition, because it is present in germline cells, at least one integrated copy of the HHV-6 genome is presumed to be present in every nucleated cell. Both HHV-6A and HHV-6B can integrate into the chromosomes; of 34 published examples for which integration sites were mapped, 9 (26%) were HHV-6A and 25 (74%) were HHV-6B (all 10 from Japan reported HHV-6B). In addition to genetic transmission, populations of cells harboring ciHHV-6 may be transmitted via allogenic hematopoietic stem cell transplantation (HSCT) 42 43 44 and probably via solid organ transplantation (SOT). HHV-6 is the only human herpesvirus known to be integrated into germline chromosomal telomeres. However, Marek's Disease virus, a herpesvirus of chickens, integrates into chromosomes by a molecular mechanism similar to HHV-6 2003, and EBV can integrate into non-telomeric regions of chromosomes in virally transformed cell lines and Burkitt lymphoma 2006.

The prevalence of ciHHV-6 is ∼1% in umbilical cord bloods and in healthy blood donors from the USA and UK, 0.2% in hospitalized patients from Japan, and ∼2% in patient groups from the USA and UK (Table).

**Table 5:** ciHHV-6 prevalence in control and patient populations in Europe, the UK and the United States

Study population	Country	ciHHV-6	*n*	%	References
**Blood donors and cord blood**					
Cord blood	USA	48	5638	0.85%	[58,77]
Blood donors	UK	4	500	0.80%	2007
Blood donors	USA	1	100	1.00%	2008
**Totals**		**53**	**6238**	**0.85**% [9]	
**Patient groups**					
Anonymous children's sera bank	UK	10	653	1.53%	2005
Liver transplant patients	US	7	548	1.28%	2011
Liver transplant patients	UK	3	60	5.00%	1999
Pediatric ALL and myeloid leukemia	Czech Rep	5	339	1.47%	2009
HSCT recipients	USA	6	322	1.86%	2011
AlloSCT recipients	Italy	1	70	1.43%	2009
Children referred for possible encephalitis	UK, Ireland	6	184	3.26%	2005
Solid organ transplant recipients	Italy	1	135	0.74%	2009
Non-Hodgkin's lymphoma	Italy	2	64	3.13%	1995
Hodgkin's disease	Italy	7	55	12.73%	1995
Kidney transplant patients	UK	1	52	1.92%	2000
Kidney transplant patients	USA	1	47	2.13%	2011
Multiple sclerosis patients	Italy	1	35	2.86%	1995
**Totals**		**51**	**2564**	**1.99**% [9]	

A chi-square significance test was conducted to determine if these prevalence data are significantly different on a statistical level. The test yielded a *p*-value of <0.001 when comparing the blood donor and cord blood group with the combined patient population.

ciHHV-6, chromosomally integrated human herpesvirus 6; HSCT, hematopoietic stem cell transplantation; ALL, acute lymphocytic leukemia; AlloSCT, allogeneic stem cell transplant.

### Why is it important to identify individuals with chromosomally integrated human herpesvirus 6?

Identifying individuals with ciHHV-6 is important because every cell in their body harbors the complete HHV-6 genome covalently linked to human chromosomal DNA. Therefore, clinical specimens from such individuals (e.g., whole blood, leukocytes, plasma, and tissue specimens) will contain HHV-6 DNA when tested by PCR assays and will be reported to have high levels of HHV-6 DNA when tested by quantitative PCR. Considering that peripheral blood contains between 4 and 7 million leukocytes (and a corresponding number of viral genomes) per ml, quantitative PCR results for HHV-6 will usually be greater than 1 × 10^6^ HHV-6 genomes/ml of whole blood. In addition, body fluids that are expected to harbor only small numbers of cells (e.g., serum, plasma, and CSF) will often be positive for HHV-6 DNA by PCR, albeit at lower copy numbers compared with cellular samples, as they contain DNA that has been released by cell lysis due to natural processes or during specimen transport and processing.

The high viral DNA load in patients with ciHHV-6 can lead to misdiagnosis due to the incorrect assumption that the patient is experiencing active HHV-6 infection for which antiviral treatment might be considered 2007. Furthermore, some drugs and chemicals have been shown to induce replication of ciHHV-6 in cell culture (Table) 2010. These activities were seen at drug concentrations comparable with levels achieved during therapeutic use. Although there is no evidence that it occurs, clinical use of these or related drugs might activate the virus in patients with ciHHV-6, with unknown clinical consequences. One study of 548 solid organ transplant recipients suggested that those with ciHHV-6 may be at increased risk for bacterial infection and graft rejection 2011.

### When should chromosomally integrated human herpesvirus 6 screening take place?

No pathology has been conclusively associated with ciHHV-6, thus routine screening is not recommended. However, in certain clinical situations, screening for ciHHV-6 should be considered, such as when there is clinical suspicion for HHV-6 reactivation and the knowledge of the patient's ciHHV-6 status would influence treatment decisions. For example, ciHHV-6 screening of patients who have high DNA copy numbers may prevent the unnecessary use of antiviral treatment. Testing may also be considered for patients who have had an adverse reaction to a drug previously shown to be associated with HHV-6 reactivation (Table).

### Can chromosomally integrated human herpesvirus 6 replicate as a virus?

Although triggers for activation of ciHHV-6 *in vivo* have not been identified, there are suggestions that ciHHV-6 can be induced to a state of lytic (active) viral replication. ciHHV-6 present in cultured lymphocytes of individuals with ciHHV-6 can be induced to lytic replication by histone deacetylase (HDAC) inhibitors, compounds known to reactivate other herpesviruses from latency 2010. Marek's Disease virus can reactivate to lytic replication from its integrated state *in vitro* [45,49]. HHV-6 DNA has been detected in the cord blood and saliva of non-ciHHV-6 children born to ciHHV-6 mothers, suggesting the possibility of transplacental transmission of free virus 2010.

### Which drugs or chemicals might lead to human herpesvirus 6 lytic replication in individuals with chromosomally integrated human herpesvirus 6?

Although evidence is lacking, it is possible that treatment with or exposure to certain pharmaceuticals or chemicals can either directly or indirectly reactivate ciHHV-6. As noted, the HDAC inhibitor Trichostatin A can reactivate HHV-6 *in vitro* in lymphocytes from individuals with ciHHV-6 2010, and two commonly used pharmaceuticals can enhance HHV-6 replication *in vitro* and *in vivo* [51,52] (Table). HHV-6 reactivation has been detected by serology and PCR in a high percentage (62%–100%) of patients with DIHS and is also frequently reported in patients with DRESS [21,53]. The mechanism of HHV-6 reactivation during DRESS/DIHS is unknown, but the drugs that activate the virus in these diseases might also activate the virus in individuals with ciHHV-6.

### Should certain drugs be avoided in individuals with chromosomally integrated human herpesvirus 6?

It is not known whether ciHHV-6 individuals are put at clinical risk by the use of drugs that have been associated with HHV-6 reactivation *in vivo* or *in vitro*. Nonetheless, we urge careful observation when use of such drugs is indicated in individuals known to have ciHHV-6.

### What is the best way to identify individuals with chromosomally integrated human herpesvirus 6?

When plasma or serum HHV-6 PCR levels are suspiciously high, the most practical way to confirm that a patient has ciHHV-6 is by quantitative PCR using whole blood or isolated PBMC's. Individuals with ciHHV-6 have significantly higher viral DNA loads in PBMC's and whole blood than do non-ciHHV-6 individuals, even those with primary HHV-6 infection 2010. By quantitative PCR, most healthy adult blood donors have low to undetectable HHV-6 DNA in their whole blood, and in one study of 496 UK blood donors, <2% had HHV-6 DNA levels in the range of 3.2–3.5 log_10_ DNA copies/ml of whole blood 2007. In contrast, individuals with ciHHV-6 have one or more HHV-6 genomic copies per white blood cell, which corresponds to >5.5 log_10_ copies/ml of whole blood 43 54 56, and the high viral DNA loads persist over time 55 57 58. In contrast, transplant recipients with HHV-6 reactivation and children with primary HHV-6B infection typically have transient virus DNA loads between 1.5 and 5.0 log_10_ copies/ml in whole blood or PBMC's and less than 5.0 log_10_ copies/ml in serum, respectively 54 59 60. Rarely, allogeneic HSCT patients with graft-versus-host disease (GVHD) and patients with DIHS/DRESS have been reported to have transient levels >6.0 log_10_ copies/ml in serum and plasma 2010. In contrast to transient viral elevations in these patients, the very high levels of HHV-6 in ciHHV-6 patients are persistent. Thus, if a patient has >5.5 log_10_ copies/ml in whole blood, ciHHV-6 should be considered, and a confirmatory test is recommended (see preceding text).

In individuals with ciHHV-6, the HHV-6 DNA load in blood will vary according to the number of cells included in the specimen, so it is essential to also consider the ratio of viral DNA copies to copies of cellular DNA, especially when the patient has leukopenia or leukocytosis 2009 or when testing body fluids such as CSF.

### Can serum or plasma polymerase chain reaction be used to identify and monitor chromosomally integrated human herpesvirus 6?

Although measuring HHV-6 DNA loads in plasma is widely accepted as a reliable marker of active HHV-6 infection in transplant recipients and other patients, virus monitoring in plasma and serum is unreliable both for identifying and for monitoring subjects with ciHHV-6. Individuals without ciHHV-6 who are experiencing active HHV-6 infections (e.g., during primary infection) sometimes have serum levels as high or higher than routinely seen in individuals with ciHHV-6, although these levels are not persistent. Monitoring ciHHV-6 individuals using plasma is problematic because quantitative PCR results from plasma can vary depending on how quickly the specimen is processed and the limit of detection of the assay. Cell lysis increases as a function of elapsed time between the blood collection and centrifugation, and the extent of lysis can be influenced by factors such as storage temperature and physical forces. Cell lysis during overnight shipping can result in plasma DNA loads in specimens from individuals with ciHHV-6 being significantly higher than a sample from the same blood draw that was separated within a few hours of venipuncture. Plasma samples from individuals with ciHHV-6 centrifuged immediately after a blood draw can test negative for HHV-6 DNA. The clotting process during serum preparation lyses cells and releases cellular DNA, resulting in serum levels of HHV-6 DNA as much as 100-fold higher than in plasma 2006.

### Will cerebrospinal fluid from individuals with chromosomally integrated human herpesvirus 6 contain human herpesvirus 6 DNA?

There are no data for HHV-6 DNA levels in the CSF of healthy ciHHV-6 patients. Individuals with ciHHV-6 who have acute neurological presentations including herpes simplex virus and EBV meningoencephalitis may have high CSF HHV-6 DNA loads (mean 4.0 log_10_ copies/ml) if there is pleocytosis 2007. Because normal CSF may contain up to five nucleated cells per μL, HHV-6 PCR can be positive when using a sensitive assay. However, under circumstances where CSF is relatively acellular, an HHV-6 DNA PCR test could be negative, especially if the assay is insensitive (cutoff >200 copies/ml). Determining the number of viral DNA copies per leukocyte may be helpful.

### Where can physicians send specimens for chromosomally integrated human herpesvirus 6 testing?

Several commercial and hospital or university laboratories offer whole blood testing for HHV-6 by quantitative PCR in the USA, Europe, and Japan. Reference lists of commercial sources for HHV-6 testing are available [5,64].

### Can whole blood quantitative polymerase chain reaction results be used to monitor chromosomally integrated human herpesvirus 6 patients?

In HSCT or SOT recipients, the presence of ciHHV-6 complicates the interpretation of quantitative PCR data because the amount of additional HHV-6 DNA resulting from active replication is most likely a small fraction of the amount that would already be present at baseline due to the ciHHV-6.

There is no demonstrated value for routinely monitoring HHV-6 DNA levels in individuals with ciHHV-6, the precision of quantitation at such high copy numbers has not been documented, and there is no proven framework for interpreting such data.

### Is it necessary to test hair follicles or nails or to perform fluorescence in situ hybridization to confirm chromosomally integrated human herpesvirus 6?

No. Whole blood quantitative PCR testing provides a high level of certainty as to whether an individual has ciHHV-6. Confirmation can be made by testing the patient's parents or siblings, or sequential testing of the patient to demonstrate persistence of high HHV-6 DNA. For ciHHV-6, which is passed through the germline, at least one biological parent would carry ciHHV-6.

DNA PCR testing of hair follicles or nails can confirm ciHHV-6 status, because only ciHHV-6 individuals have detectable HHV-6 DNA in these tissues [57,65]. These assays might be useful when obtaining blood samples is complicated, particularly in developing countries. Fluorescence in situ hybridization analysis can also establish probable integration, but such assays are complex and are not necessary for diagnostic purposes given the availability of quantitative PCR testing.

Identification of ciHHV-6 can be considered as a form of genetic testing that can unintentionally illuminate issues related to biological parentage (e.g., a child is positive for ciHHV-6 and both parents are negative). Consultation with a genetic counselor might be warranted.

### Do individuals with chromosomally integrated human herpesvirus 6 produce antibodies against human herpesvirus 6 antigens?

Antibodies against HHV-6 proteins might be generated against viral proteins expressed from an otherwise quiescent chromosomally integrated virus genome during latency, during lytic reactivation of the chromosomally integrated genome, and during infection with a community acquired virus. Little is known about whether and how the chromosomally integrated virus might affect the immune response during active infections, for example, whether immune tolerance develops for some viral proteins. In one study, it appeared that antibody levels among those with ciHHV-6 might be reduced; only 14% of ciHHV-6 individuals had antibodies to HHV-6 glycoprotein B, compared with 60% without ciHHV-6 2004.

### How can you tell if an individual with chromosomally integrated human herpesvirus 6 has active human herpesvirus 6 infection?

A single quantitative PCR test on serum or plasma cannot prove whether a patient with ciHHV-6 has active HHV-6 infection, whether due to activation of ciHHV-6 or horizontally acquired. Neither serum nor plasma PCR is definitive for this purpose. Quantitative reverse transcriptase PCR that monitors the expression of HHV-6 genes associated with productive infection (e.g., structural genes) might be useful for the identification of active infections, but such assays are not generally available and their clinical utility for monitoring HHV-6 activity in individuals with ciHHV-6 has not been defined. Alternatively, active HHV-6 infections can be detected using a quantitative antigenemia assay that measures the frequency of peripheral blood leukocytes that express lytic antigens [67,68], but such an assay has not been evaluated in the context of ciHHV-6. Physicians must, therefore, use clinical judgment to determine if the patient is experiencing a disease typically associated with HHV-6.

### What treatments are effective for human herpesvirus 6 infections?

No drugs have been specifically approved for HHV-6 infections by government agencies, and no large trials have confirmed that antivirals are effective against HHV-6 *in vivo*. Several small studies have demonstrated that prophylactic antiviral treatment inhibits reactivation and may reduce the number of neurological events in HSCT patients 69 70 71. Although most strains of HHV-6A and HHV-6B are susceptible to both ganciclovir and foscarnet, some HHV-6B strains are resistant to ganciclovir 2009. Neither acyclovir nor penciclovir are effective against either HHV-6A or HHV-6B. Ganciclovir, foscarnet, and cidofovir are approved for treatment of the closely related betaherpesvirus CMV and have been used to treat HHV-6 diseases such as encephalitis in the post-transplant setting 68 72 73. Valganciclovir decreased the incidence of HHV-6 viremia in SOT recipients, and ganciclovir prophylaxis delayed and shortened HHV-6 viremia in renal transplant recipients. Ganciclovir treatment of CMV disease in liver transplant patients reduced concurrent HHV-6 antigenemia, although the response was slower than for CMV 2002. HHV-6 infections in allogeneic HSCT patients have been successfully treated with foscarnet 68 69 75. In immunocompromised hosts, reduction in pharmacologic immunosuppression to improve T cell function is an important and often essential component of therapy for HHV-6.

### Should patients with chromosomally integrated human herpesvirus 6 be treated for human herpesvirus 6?

There is no basis to recommend treatment of asymptomatic individuals with ciHHV-6 for their resident viral DNA 2008. It is not known whether individuals with ciHHV-6 are more prone to develop active HHV-6 infections or whether such hypothetical infections cause any disease. Nevertheless, antiviral therapy might be warranted if patients present with clinical manifestations typically associated with reactivated HHV-6 infection. In all instances, alternative and concurrent causes of the clinical manifestations should be sought, such as CMV and other herpesviruses, even when the clinical symptoms are consistent with those associated with HHV-6. Although some ciHHV-6 patients with encephalitis and encephalomyelitis [21,52] have appeared to respond to antiviral treatment, in other cases, the therapy was ineffective [46,76].

### How should chromosomally integrated human herpesvirus 6 families be advised on pregnancy?

Little is known about ciHHV-6 and pregnancy 2010. No published studies have suggested a reason to avoid pregnancy or that ciHHV-6 individuals bear an increased risk of developing specific diseases.

### Should individuals with chromosomally integrated human herpesvirus 6 refrain from donating blood, stem cells, ova, sperm, or organs?

Case reports of HSCT from ciHHV-6 positive individuals to negative recipients suggest that ciHHV-6 individuals can successfully donate [43,44]. However, only a handful of such cases have been studied. The possibility that donated blood, blood products, cells, or tissues from individuals with ciHHV-6 might cause clinical problems requires further study.

**Key points**

- Approximately 1% of the population harbors germline ciHHV-6.
- Because it is transmitted via the germline, the HHV-6 genome is present in every nucleated cell in the body.
- HHV-6 levels in whole blood >5.5 log_10_ copies/ml are suggestive of ciHHV-6.
- ciHHV-6 transplant patients may be more likely to experience GVHD and bacterial infections.
- Integrated HHV-6 can be activated *in vitro*.
- ciHHV-6 can lead to the misdiagnosis of reactivated HHV-6 infection.

## Abbreviations

AHS: anticonvulsant-induced hypersensitiviy syndrome
ALL: acute lymphocytic leukemia
DIHS: drug-induced hypersensitivity syndrome
DRESS: drug rash with eosinophilia and systemic symptoms
FDA: United States' Food and Drug Administration
FISH: fluorescence in situ hybridization
GVHD: graft-versus-host disease
HDAC: histone deacetylase
HSCT: hematopoietic stem cell transplantation
SJS: Stevens–Johnson syndrome
SOT: solid organ transplantation
TEN: toxic epidermal necrolysis

## References

[1] Clark DA, Ward KN (2008). Importance of chromosomally integrated HHV-6A and -6B in the diagnosis of active HHV-6 infection. Herpes.

[2] Yamanishi K, Mori M, Pellett PE, David PMH, Knipe M (2007). Human herpesvirus 6 and 7. Fields Virology.

[3] Morissette G, Flamand L (2010). Herpesviruses and chromosomal integration. Journal of Virology.

[4] Arbuckle JH, Medveczky PG (2011). The molecular biology of human herpesvirus-6 latency and telomere integration. Microbes Infect.

[5] Pellett PE, T.G., F.G., Versalovic KCC, Jorgensen JH, Landry ML, Warnock DW (2011). Human herpesviruses 6, 7, and 8., in Manual of Clinical Microbiology.

[6] Lee SO, Brown RA, Razonable RR (2011). Clinical significance of pretransplant chromosomally integrated human herpesvirus-6 in liver transplant recipients. Transplantation.

[7] Yamashita N, Morishima T (2005). HHV-6 and seizures. Herpes.

[8] Ichiyama T (2009). Serum and cerebrospinal fluid levels of cytokines in acute encephalopathy associated with human herpesvirus-6 infection. Brain & Development.

[9] Seeley WW, Marty FM, Holmes TM (2007). Post-transplant acute limbic encephalitis: clinical features and relationship to HHV6. Neurology.

[10] Gorniak RJ, Young GS, Wiese DE, Marty FM, Schwartz RB (2006). MR imaging of human herpesvirus-6-associated encephalitis in 4 patients with anterograde amnesia after allogeneic hematopoietic stem-cell transplantation. AJNR. American Journal of Neuroradiology.

[11] Bollen AE, Wartan AN, Krikke AP, Haaxma-Reiche H (2001). Amnestic syndrome after lung transplantation by human herpes virus-6 encephalitis. Journal of Neurology.

[12] Zerr DM, Fann JR, Breiger D (2011). HHV-6 reactivation and its effect on delirium and cognitive functioning in hematopoietic cell transplantation recipients. Blood.

[13] Maric I, Bryant R, Abu-Asab M (2004). Human herpesvirus-6-associated acute lymphadenitis in immunocompetent adults. Modern Pathology.

[14] Revest M, Minjolle S, Veyer D, Lagathu G, Michelet C, Colimon R (2011). Detection of HHV-6 in over a thousand samples: new types of infection revealed by an analysis of positive results. Journal of Clinical Virology.

[15] Lamoth F, Jayet PY, Aubert JD (2008). Case report: human herpesvirus 6 reactivation associated with colitis in a lung transplant recipient. Journal of Medical Virology.

[16] Amo K, Tanaka-Taya K, Inagi R (2003). Human herpesvirus 6B infection of the large intestine of patients with diarrhea. Clinical Infectious Diseases.

[17] Chevret L, Boutolleau D, Halimi-Idri N (2008). Human herpesvirus-6 infection: a prospective study evaluating HHV-6 DNA levels in liver from children with acute liver failure. Journal of Medical Virology.

[18] Yoshikawa T (2006). Human herpesvirus 6 causes hepatitis in transplant recipients. Internal Medicine.

[19] Peppercorn AF, Miller MB, Fitzgerald D, Weber DJ, Groben PA, Cairns BA (2010). High-level human herpesvirus-6 viremia associated with onset of Stevens-Johnson syndrome: report of two cases. Journal of Burn Care & Research.

[20] Chapenko S, Folkmane I, Ziedina I (2009). Association of HHV-6 and HHV-7 reactivation with the development of chronic allograft nephropathy. Journal of Clinical Virology.

[21] Tohyama M, Hashimoto K, Yasukawa M (2007). Association of human herpesvirus 6 reactivation with the flaring and severity of drug-induced hypersensitivity syndrome. British Journal of Dermatology.

[22] Marabelle A, Bergeron C, Billaud G, Mekki Y, Girard S (2010). Hemophagocytic syndrome revealing primary HHV-6 infection. Journal de Pediatria.

[23] Dharancy S, Crombe V, Copin MC (2008). Fatal hemophagocytic syndrome related to human herpesvirus-6 reinfection following liver transplantation: a case report. Transplantation Proceedings.

[24] Kuhl U, Pauschinger M, Noutsias M (2005). High prevalence of viral genomes and multiple viral infections in the myocardium of adults with " idiopathic" left ventricular dysfunction. Circulation.

[25] Leveque N, Boulagnon C, Brasselet C (2011). A fatal case of human herpesvirus 6 chronic myocarditis in an immunocompetent adult. Journal of Clinical Virology.

[26] Mahrholdt H, Wagner A, Deluigi CC (2006). Presentation, patterns of myocardial damage, and clinical course of viral myocarditis. Circulation.

[27] Yamaguchi N, Takatsuka H, Wakae T (2003). Idiopathic interstitial pneumonia following stem cell transplantation. Clinical Transplantation.

[28] Cone RW, Hackman RC, Huang ML (1993). Human herpesvirus 6 in lung tissue from patients with pneumonitis after bone marrow transplantation. The New England Journal of Medicine.

[29] Hammerling JA, Lambrecht RS, Kehl KS, Carrigan DR (1996). Prevalence of human herpesvirus 6 in lung tissue from children with pneumonitis. Journal of Clinical Pathology.

[30] Kano Y, M Inaoka, Shiohara T (2004). Association between anticonvulsant hypersensitivity syndrome and human herpesvirus 6 reactivation and hypogammaglobulinemia. Archives of Dermatology.

[31] Takatsuka H, Wakae T, Mori A (2003). Endothelial damage caused by cytomegalovirus and human herpesvirus-6. Bone Marrow Transplantation.

[32] Matsuda Y, Hara J, Miyoshi H (1999). Thrombotic microangiopathy associated with reactivation of human herpesvirus-6 following high-dose chemotherapy with autologous bone marrow transplantation in young children. Bone Marrow Transplantation.

[33] Martinez A, Alvarez-Lafuente R, Mas A (2007). Environment-gene interaction in multiple sclerosis: human herpesvirus 6 and MHC2TA. Human Immunology.

[34] Garcia-Montojo M, De Las Heras V, Dominguez-Mozo M (2011). Human herpesvirus 6 and effectiveness of interferon beta 1b in multiple sclerosis patients. European Journal of Neurology.

[35] Lusso P, Crowley RW, Malnati MS (2007). Human herpesvirus 6Aaccelerates AIDS progression in macaques. Proceedings of the National Academy of Sciences of the United States of America.

[36] Lusso P, Gallo RC (1995). Human herpesvirus 6 in AIDS. Immunology Today.

[37] Emery VC, Atkins MC, Bowen EF (1999). Interactions between beta-herpesviruses and human immunodeficiency virus in vivo: evidence for increased human immunodeficiency viral load in the presence of human herpesvirus 6. Journal of Medical Virology.

[38] Crawford JR, Kadom N, Santi MR, Mariani B, Lavenstein BL (2007). Human herpesvirus 6 rhombencephalitis in immunocompetent children. Journal of Child Neurology.

[39] Potenza L, Luppi M, Barozzi P (2008). HHV-6A in syncytial giant-cell hepatitis. The New England Journal of Medicine.

[40] Ablashi DV, Devin CL, Yoshikawa T (2010). Review part 3: human herpesvirus-6 in multiple non-neurological diseases. Journal of Medical Virology.

[41] Yao K, Crawford JR, Komaroff AL, Ablashi DV, Jacobson S (2010). Review part 2: human herpesvirus-6 in central nervous system diseases. Journal of Medical Virology.

[42] Luppi M, Barozzi P, Bosco R (2006). Human herpesvirus 6 latency characterized by high viral load: chromosomal integration in many, but not all, cells. Journal of Infectious Diseases.

[43] Clark DA, Nacheva EP, Leong HN (2006). Transmission of integrated human herpesvirus 6 through stem cell transplantation: implications for laboratory diagnosis. Journal of Infectious Diseases.

[44] Kamble RT, Clark DA, Leong HN, Heslop HE, Brenner MK, Carrum G (2007). Transmission of integrated human herpesvirus-6 in allogeneic hematopoietic stem cell transplantation. Bone Marrow Transplantation.

[45] Parcells MS, Arumugaswami V, Prigge JT, Pandya K, Dienglewicz RL (2003). Marek's disease virus reactivation from latency: changes in gene expression at the origin of replication. Poultry Science.

[46] Reisinger J, Rumpler S, Lion T, Ambros PF (2006). Visualization of episomal and integrated Epstein-Barr virus DNA by fiber fluorescence in situ hybridization. International Journal of Cancer.

[47] Hubacek P, Maalouf J, Zajickova M (2007). Failure of multiple antivirals to affect high HHV-6 DNAaemia resulting from viral chromosomal integration in case of severe aplastic anaemia. Haematologica.

[48] Arbuckle JH, Medveczky MM, Luka J (2010). The latent human herpesvirus-6A genome specifically integrates in telomeres of human chromosomes in vivo and in vitro. Proceedings of the National Academy of Sciences of the United States of America.

[49] Delecluse HJ, W Hammerschmidt (1993). Status of Marek's disease virus in established lymphoma cell lines: herpesvirus integration is common. Journal of Virology.

[50] Hall CB, Caserta MT, Schnabel KC (2010). Transplacental congenital human herpesvirus 6 infection caused by maternal chromosomally integrated virus. Journal of Infectious Diseases.

[51] Mardivirin L, Valeyrie-Allanore L, Branlant-Redon E (2010). Amoxicillin-induced flare in patients with DRESS (Drug Reaction with Eosinophilia and Systemic Symptoms): report of seven cases and demonstration of a direct effect of amoxicillin on human herpesvirus 6 replication in vitro. European Journal of Dermatology.

[52] Mardivirin L, Descamps V, Lacroix A, Delebassee S, Ranger-Rogez S (2009). Early effects of drugs responsible for DRESS on HHV-6 replication in vitro. Journal of Clinical Virology.

[53] Eshki M, Allanore L, Musette P (2009). Twelve-year analysis of severe cases of drug reaction with eosinophilia and systemic symptoms: a cause of unpredictable multiorgan failure. Archives of Dermatology.

[54] Caserta MT, Hall CB, Schnabel K (2010). Diagnostic assays for active infection with human herpesvirus 6 (HHV-6). Journal of Clinical Virology.

[55] Leong HN, Tuke PW, Tedder RS (2007). The prevalence of chromosomally integrated human herpesvirus 6 genomes in the blood of UK blood donors. Journal of Medical Virology.

[56] Deback C, Geli J, Ait-Arkoub Z (2009). Use of the Roche LightCycler 480 system in a routine laboratory setting for molecular diagnosis of opportunistic viral infections: evaluation on whole blood specimens and proficiency panels. Journal of Virological Methods.

[57] Ward KN, Leong HN, Nacheva EP (2006). Human herpesvirus 6 chromosomal integration in immunocompetent patients results in high levels of viral DNA in blood, sera, and hair follicles. Journal of Clinical Microbiology.

[58] Hall CB, Caserta MT, Schnabel K (2008). Chromosomal integration of human herpesvirus 6 is the major mode of congenital human herpesvirus 6 infection. Pediatrics.

[59] Ogata M, Kikuchi H, Satou T (2006). Human herpesvirus 6 DNA in plasma after allogeneic stem cell transplantation: incidence and clinical significance. Journal of Infectious Diseases.

[60] Secchiero P, Carrigan DR, Asano Y (1995). Detection of human herpesvirus 6 in plasma of children with primary infection and immunosuppressed patients by polymerase chain reaction. Journal of Infectious Diseases.

[61] Brands-Nijenhuis AV, van Loo IH, Schouten HC, van Gelder M (2010). Temporal relationship between HHV 6 and graft vs host disease in a patient after haplo-identical SCT and severe T-cell depletion. Bone Marrow Transplant.

[62] Gautheret-Dejean A, Henquell C, Mousnier F (2009). Different expression of human herpesvirus-6 (HHV-6) load in whole blood may have a significant impact on the diagnosis of active infection. Journal of Clinical Virology.

[63] Ward KN, Leong HN, Thiruchelvam AD, Atkinson CE, Clark DA (2007). Human herpesvirus 6 DNA levels in cerebrospinal fluid due to primary infection differ from those due to chromosomal viral integration and have implications for diagnosis of encephalitis. Journal of Clinical Microbiology.

[64] HHV-6-Foundation http://www.hhv-6foundation.org/clinicians/cihhv-6-testing.

[65] Hubacek P, Virgili A, Ward KN (2009). HHV-6 DNA throughout the tissues of two stem cell transplant patients with chromosomally integrated HHV-6 and fatal CMV pneumonitis. British Journal of Haematology.

[66] Tanaka-Taya K, Sashihara J, Kurahashi H (2004). Human herpesvirus 6 (HHV-6) is transmitted from parent to child in an integrated form and characterization of cases with chromosomally integrated HHV-6 DNA. Journal of Medical Virology.

[67] Loginov R, Karlsson T, Hockerstedt K, Ablashi D, Lautenschlager I (2010). Quantitative HHV-6B antigenemia test for the monitoring of transplant patients. European Journal of Clinical Microbiology and Infectious Diseases.

[68] Flamand L, Komaroff AL, Arbuckle JH, Medveczky PG, Ablashi DV (2010). Review, part 1: human herpesvirus-6-basic biology, diagnostic testing, and antiviral efficacy. Journal of Medical Virology.

[69] Ishiyama K, Katagiri T, Ohata K (2011). Safety of pre-engraftment prophylactic foscarnet administration after allogeneic stem cell transplantation. Transpl Infect Dis.

[70] Tokimasa S, Hara J, Osugi Y (2002). Ganciclovir is effective for prophylaxis and treatment of human herpesvirus-6 in allogeneic stem cell transplantation. Bone Marrow Transplantation.

[71] Rapaport D, Engelhard D, Tagger G, Or R, Frenkel N (2002). Antiviral prophylaxis may prevent human herpesvirus-6 reactivation in bone marrow transplant recipients. Transplant Infectious Disease.

[72] Razonable RR, Zerr DM (2009). HHV-6, HHV-7 and HHV-8 in solid organ transplant recipients. American Journal of Transplantation.

[73] Zerr DM, Gupta D, Huang ML, Carter R, Corey L (2002). Effect of antivirals on human herpesvirus 6 replication in hematopoietic stem cell transplant recipients. Clinical Infectious Diseases.

[74] Lautenschlager I, Lappalainen M, Linnavuori K, Suni J, Hockerstedt K (2002). CMV infection is usually associated with concurrent HHV-6 and HHV-7 antigenemia in liver transplant patients. Journal of Clinical Virology.

[75] Ishiyama K, Katagiri T, Hoshino T, Yoshida T, Yamaguchi M, Nakao S (2010). Preemptive therapy of human herpesvirus-6 encephalitis with foscarnet sodium for high-risk patients after hematopoietic SCT. Bone Marrow Transplant.

[76] Ljungman P, de la Camara R, Cordonnier C (2008). Management of CMV, HHV-6, HHV-7 and Kaposi-sarcoma herpesvirus (HHV-8) infections in patients with hematological malignancies and after SCT. Bone Marrow Transplantation.

[77] Hall CB, Caserta MT, Schnabel KC (2004). Congenital infections with human herpesvirus 6 (HHV6) and human herpesvirus 7 (HHV7). Journal de Pediatria.

[78] Hudnall SD, Chen T, Allison P, Tyring SK, Heath A (2008). Herpesvirus prevalence and viral load in healthy blood donors by quantitative real-time polymerase chain reaction. Transfusion.

[79] Ward KN, Thiruchelvam AD, Couto-Parada X (2005). Unexpected occasional persistence of high levels of HHV-6 DNA in sera: detection of variants A and B. Journal of Medical Virology.

[80] Lee SO, Brown RA, Razonable RRl (2011). Clinical Significance of Pretransplant Chromosomally Integrated Human Herpesvirus-6 in Liver Transplant Recipients. Transplantation.

[81] Griffiths PD, Ait-Khaled M, Bearcroft CP (1999). Human herpesviruses 6 and 7 as potential pathogens after liver transplant: prospective comparison with the effect of cytomegalovirus. Journal of Medical Virology.

[82] Hubacek P, Muzikova K, Hrdlickova A (2009). Prevalence of HHV-6 integrated chromosomally among children treated for acute lymphoblastic or myeloid leukemia in the Czech Republic. Journal of Medical Virology.

[83] Potenza L, Barozzi P, Masetti M (2009). Prevalence of human herpesvirus-6 chromosomal integration (CIHHV-6) in Italian solid organ and allogeneic stem cell transplant patients. American Journal of Transplantation.

[84] Ward KN (2005). The natural history and laboratory diagnosis of human herpesviruses-6 and −7 infections in the immunocompetent. Journal of Clinical Virology.

[85] Torelli G, Barozzi P, Marasca R (1995). Targeted integration of human herpesvirus 6 in the p arm of chromosome 17 of human peripheral blood mononuclear cells in vivo. Journal of Medical Virology.

[86] Kidd IM, Clark DA, Sabin CA (2000). Prospective study of human betaherpesviruses after renal transplantation: association of human herpesvirus 7 and cytomegalovirus co-infection with cytomegalovirus disease and increased rejection. Transplantation.

[87] Lee SO, Brown RA, Eid AJ, Razonable RR (2011). Chromosomally integrated human herpesvirus-6 in kidney transplant recipients. Nephrology, Dialysis, Transplantation.

