# Supplementary material for: Chromosomally integrated human herpesvirus 6: questions and answers
Source: Rev Med Virol. 2011 Nov 4;22(3):144–55. doi: 10.1002/rmv.715 (PMC3498727; doi:10.1002/rmv.715)
Supplement: Supplementary file 1 [file rmv0022-0144-sd1.doc]

**Supplemental Table S1. Additional information and references related to Table 4.**

***Drugs associated with HHV-6 reactivation in vivo in DIHS, DRESS, AHS, SJS, and TEN***

| **Drug** | **Associations** | **References** | **Notes** |
| --- | --- | --- | --- |
| **IN VIVO** |  |  |  |
| allopurinol | DIHS, DRESS, SJS | [^1-7^](#_ENREF_1) | Enzyme inhibitor used for gout; may cause a drop in circulating B cells [^8^](#_ENREF_8). |
| carbamazepine | AHS, DIHS, DRESS, SJS, TEN |  | Anticonvulsant and HDAC inhibitor. Associated with transient hypogammaglobulinemia . |
| dapsone | AHS, DIHS, DRESS |  | Antibiotic and anti-malarial drug. |
| ibuprofen | DIHS, DRESS |  | Non-steroidal anti-inflammatory drug; one case was in association with diclofenac [^23^](#_ENREF_23). |
| lamotrigine | AHS, DIHS, DRESS |  | Anticonvulsant. |
| mexiletine | DIHS |  | Class 1B anti-arrhythmic. |
| minocycline | DIHS, DRESS |  | Broad spectrum tetracycline antibiotic. |
| naproxen | DRESS | [^30^](#_ENREF_30) | Non-steroidal anti-inflammatory drug that inhibits both COX-1 and COX-2 enzymes. |
| phenytoin | AHS, DIHS |  | Cross-sensitive with carbamazepine and phenobarbital [^18^](#_ENREF_18). |
| phenobarbital | AHS, DIHS, DRESS |  | Barbituate and anticonvulsant known to induce EBV in Raji cells; cross-sensitive with carbamazepine and phenytoin [^18^](#_ENREF_18). |
| sodium valproate | AHS, DIHS | [^38^](#_ENREF_38) | Anticonvulsant, HDAC inhibitor and rare cause of DIHS. Three cases have been reported, but in two of them HHV-6 was not tested . |
| sulfasalazine | DIHS, DRESS, |  | Anti-inflammatory that is not an immunosuppressant; used in inflammatory bowel disease and rheumatoid arthritis. |
| trichloroethylene | DIHS |  | HDAC inhibitor and industrial solvent previously used as an anesthetic and inhaled obstetrical analgesic. |
| trimethoprim-sulfamethoxazole | DIHS, DRESS |  | Sulfonamide antibiotic. |
| vancomycin | DRESS |  | In one case teicoplanin was also reactive [^49^](#_ENREF_49). |
| zonisamide | AHS, DIHS, TEN |  | Sulfonamide anticonvulsant. |

***Drugs associated with HHV-6 reactivation or enhanced replication*** in vitro

| **IN VITRO** | **References** | **Notes** |
| --- | --- | --- |
| [12-O-tetradecanoylphorbol-13-acetate](http://en.wikipedia.org/wiki/12-O-tetradecanoylphorbol-13-acetate)  (TPA) | [^52-54^](#_ENREF_52) | Diester of phorbol and potent tumor promoter, used in laboratories to activate herpesviruses, stimulate division of B cells, and activate signal transduction enzyme protein kinase C. |
| amoxicillin | [^5^](#_ENREF_5) | [β-lactam](http://en.wikipedia.org/wiki/Beta-lactam_antibiotic) [antibiotic](http://en.wikipedia.org/wiki/Antibiotic). |
| calcium ionophore, A23187 | [^54^](#_ENREF_54) | Ion carrier also known as calimycin or calcium ionophore; used in the laboratory to reactivate latent herpesviruses. |
| hydrocortisone |  | Cortisol, a steroid hormone produced by the adrenal gland; used in the laboratory to reactivate herpesviruses. |
| sodium n-butyrate | [^54^](#_ENREF_54) | HDAC inhibitor; used in the lab to alter gene expression or activate herpesviruses. |
| trichostatin A | [^53^](#_ENREF_53) | HDAC inhibitior; closely related Vorinostat is FDA approved for the treatment of cutaneous T cell lymphoma. |
| valproic acid and carbamazapine | [^56^](#_ENREF_56) | HDAC inhibitors; evidence conflicting because valproic acid was also shown to inhibit HHV-6B replication in one of three cell lines [^57^](#_ENREF_57). VPA inhibits EBV, but enhances HHV-8 replication in vitro [^58^](#_ENREF_58). |

***Abbreviations:***

HDAC: Histone deacetylase

DIHS: Drug Induced Hypersensitivity Syndrome
DRESS: Drug Reaction with Eosinophilia & Systemic Symptoms
AHS: Anticonvulsant syndrome
SJS: Stevens-Johnson Syndrome
TEN: Toxic Epidermal Necrolysis

**REFERENCES**

1. Suzuki Y, Inagi R, Aono T, Yamanishi K, Shiohara T. Human herpesvirus 6 infection as a risk factor for the development of severe drug-induced hypersensitivity syndrome. Arch Dermatol 1998;134:1108-12.

2. Peppercorn AF, Miller MB, Fitzgerald D, Weber DJ, Groben PA, Cairns BA. High-level human herpesvirus-6 viremia associated with onset of Stevens-Johnson syndrome: report of two cases. J Burn Care Res 2010;31:365-8.

3. Tohyama M, Hashimoto K, Yasukawa M, Kimura H, Horikawa T, Nakajima K, Urano Y, Matsumoto K, Iijima M, Shear NH. Association of human herpesvirus 6 reactivation with the flaring and severity of drug-induced hypersensitivity syndrome. Br J Dermatol 2007;157:934-40.

4. Masaki T, Fukunaga A, Tohyama M, Koda Y, Okuda S, Maeda N, Kanda F, Yasukawa M, Hashimoto K, Horikawa T, Ueda M. Human herpes virus 6 encephalitis in allopurinol-induced hypersensitivity syndrome. Acta Derm Venereol 2003;83:128-31.

5. Mardivirin L, Valeyrie-Allanore L, Branlant-Redon E, Beneton N, Jidar K, Barbaud A, Crickx B, Ranger-Rogez S, Descamps V. Amoxicillin-induced flare in patients with DRESS (Drug Reaction with Eosinophilia and Systemic Symptoms): report of seven cases and demonstration of a direct effect of amoxicillin on Human Herpesvirus 6 replication in vitro. Eur J Dermatol 2010;20:68-73.

6. Suzuki HI, Asai T, Tamaki Z, Hangaishi A, Chiba S, Kurokawa M. Drug-induced hypersensitivity syndrome with rapid hematopoietic reconstitution during treatment for acute myeloid leukemia. Haematologica 2008;93:469-70.

7. Hamaguchi Y, Fujimoto M, Enokido Y, Wayaku T, Kaji K, Echigo T, Takehara K. Intractable genital ulcers from herpes simplex virus reactivation in drug-induced hypersensitivity syndrome caused by allopurinol. Int J Dermatol 2010;49:700-4.

8. Kato C, Sato K, Wakabayashi A, Eishi Y. The effects of allopurinol on immune function in normal BALB/c and SCID mice. Int J Immunopharmacol 2000;22:547-56.

9. Teraki Y, Shibuya M, Izaki S. Stevens-Johnson syndrome and toxic epidermal necrolysis due to anticonvulsants share certain clinical and laboratory features with drug-induced hypersensitivity syndrome, despite differences in cutaneous presentations. Clin Exp Dermatol 2010;35:723-8.

10. Kano Y, Inaoka M, Shiohara T. Association between anticonvulsant hypersensitivity syndrome and human herpesvirus 6 reactivation and hypogammaglobulinemia. Arch Dermatol 2004;140:183-8.

11. Calligaris L, Stocco G, De Iudicibus S, Marino S, Decorti G, Barbi E, Carrozzi M, Marchetti F, Bartoli F, Ventura A. Carbamazepine hypersensitivity syndrome triggered by a human herpes virus reactivation in a genetically predisposed patient. Int Arch Allergy Immunol 2009;149:173-7.

12. Descamps V, Valance A, Edlinger C, Fillet AM, Grossin M, Lebrun-Vignes B, Belaich S, Crickx B. Association of human herpesvirus 6 infection with drug reaction with eosinophilia and systemic symptoms. Arch Dermatol 2001;137:301-4.

13. Sekine N, Motokura T, Oki T, Umeda Y, Sasaki N, Hayashi M, Sato H, Fujita T, Kaneko T, Asano Y, Kikuchi K. Rapid loss of insulin secretion in a patient with fulminant type 1 diabetes mellitus and carbamazepine hypersensitivity syndrome. JAMA 2001;285:1153-4.

14. Aihara Y, Ito SI, Kobayashi Y, Yamakawa Y, Aihara M, Yokota S. Carbamazepine-induced hypersensitivity syndrome associated with transient hypogammaglobulinaemia and reactivation of human herpesvirus 6 infection demonstrated by real-time quantitative polymerase chain reaction. Br J Dermatol 2003;149:165-9.

15. Zeller A, Schaub N, Steffen I, Battegay E, Hirsch HH, Bircher AJ. Drug hypersensitivity syndrome to carbamazepine and human herpes virus 6 infection: case report and literature review. Infection 2003;31:254-6.

16. Ogihara T, Takahashi T, Hanihara T, Amano N, Matsumoto K. Carbamazepine-induced hypersensitivity syndrome, associated with human herpesvirus 6 reactivation. J Clin Psychopharmacol 2004;24:105-6.

17. Nakashima H, Yamane K, Ihn H, Nakamura K, Watanabe R, Kuwano Y, Takekoshi T, Watanabe T, Hattori N, Fujimoto M, Tamaki K. Drug-induced hypersensitivity syndrome associated with transient hypogammaglobulinaemia and increase in serum IgE level. Dermatology 2005;210:349-52.

18. Oskay T, Karademir A, Erturk OI. Association of anticonvulsant hypersensitivity syndrome with Herpesvirus 6, 7. Epilepsy Res 2006;70:27-40.

19. Watanabe H, Daibata M, Tohyama M, Batchelor J, Hashimoto K, Iijima M. Chromosomal integration of human herpesvirus 6 DNA in anticonvulsant hypersensitivity syndrome. Br J Dermatol 2008;158:640-2.

20. Suzuki Y, Fukuda M, Tohyama M, Ishikawa M, Yasukawa M, Ishii E. Carbamazepine-induced drug-induced hypersensitivity syndrome in a 14-year-old Japanese boy. Epilepsia 2008;49:2118-21.

21. Watanabe T, Nakashima H, Ohmatsu H, Sakurai N, Takekoshi T, Tamaki K. Detection of human herpesvirus-6 transcripts in carbamazepine-induced hypersensitivity syndrome by in situ hybridization. J Dermatol Sci 2009;54:134-6.

22. Takahashi H, Tanaka M, Tanikawa A, Toyohara A, Ogo Y, Morimoto A, Harato R, Kobayashi M, Amagai M. A case of drug-induced hypersensitivity syndrome showing transient immunosuppression before viral reactivation during treatment for pemphigus foliaceus. Clin Exp Dermatol 2006;31:33-5.

23. Chiou CC, Chung WH, Hung SI, Yang LC, Hong HS. Fulminant type 1 diabetes mellitus caused by drug hypersensitivity syndrome with human herpesvirus 6 infection. J Am Acad Dermatol 2006;54:S14-7.

24. Gentile I, Talamo M, Borgia G. Is the drug-induced hypersensitivity syndrome (DIHS) due to human herpesvirus 6 infection or to allergy-mediated viral reactivation? Report of a case and literature review. BMC Infect Dis 2010;10:49.

25. Roquin G, Peres M, Lerolle N, Dib N, Mercat A, Croue A, Augusto JF. First report of lamotrigine-induced drug rash with eosinophilia and systemic symptoms syndrome with pancreatitis. Ann Pharmacother 2010;44:1998-2000.

26. Sekiguchi A, Kashiwagi T, Ishida-Yamamoto A, Takahashi H, Hashimoto Y, Kimura H, Tohyama M, Hashimoto K, Iizuka H. Drug-induced hypersensitivity syndrome due to mexiletine associated with human herpes virus 6 and cytomegalovirus reactivation. J Dermatol 2005;32:278-81.

27. Yagami A, Yoshikawa T, Asano Y, Koie S, Shiohara T, Matsunaga K. Drug-induced hypersensitivity syndrome due to mexiletine hydrochloride associated with reactivation of human herpesvirus 7. Dermatology 2006;213:341-4.

28. Eshki M, Allanore L, Musette P, Milpied B, Grange A, Guillaume JC, Chosidow O, Guillot I, Paradis V, Joly P, Crickx B, Ranger-Rogez S, Descamps V. Twelve-year analysis of severe cases of drug reaction with eosinophilia and systemic symptoms: a cause of unpredictable multiorgan failure. Arch Dermatol 2009;145:67-72.

29. Descamps V, Collot S, Mahe E, Houhou N, Crickx B, Ranger-Rogez S. Active human herpesvirus 6 infection in a patient with drug rash with eosinophilia and systemic symptoms. J Invest Dermatol 2003;121:215-6.

30. Pinana E, Lei SH, Merino R, Melgosa M, De La Vega R, Gonzales-Obeso E, Ramirez E, Borobia A, Carcas A. DRESS-syndrome on sulfasalazine and naproxen treatment for juvenile idiopathic arthritis and reactivation of human herpevirus 6 in an 11-year-old Caucasian boy. J Clin Pharm Ther 2010;35:365-70.

31. Fujino Y, Nakajima M, Inoue H, Kusuhara T, Yamada T. Human herpesvirus 6 encephalitis associated with hypersensitivity syndrome. Ann Neurol 2002;51:771-4.

32. Criado PR, Criado RF, Vasconcellos C, Pegas JR, Cera PC. Drug-induced hypersensitivity syndrome due to anticonvulsants in a two-year-old boy. J Dermatol 2004;31:1009-13.

33. Nagai Y, Hattori T, Ishikawa O. A case of hypersensitivity syndrome due to phenytoin. J Dermatol 2002;29:670-3.

34. Descamps V, Bouscarat F, Laglenne S, Aslangul E, Veber B, Descamps D, Saraux JL, Grange MJ, Grossin M, Navratil E, Crickx B, Belaich S. Human herpesvirus 6 infection associated with anticonvulsant hypersensitivity syndrome and reactive haemophagocytic syndrome. Br J Dermatol 1997;137:605-8.

35. Ito T, Ooishi C, Chiba A, Sakuta M, Sakuma K, Shiohara T. [Limbic encephalitis associated with drug-induced hypersensitivity syndrome due to phenobarbital--a case report]. Rinsho Shinkeigaku 2005;45:495-501.

36. Nakazato T, Suzuki K, Mihara A, Sanada Y, Aisa Y, Kakimoto T. ATL-like marked atypical lymphocytosis associated with drug-induced hypersensitivity syndrome and human herpesvirus-6 reactivation. Int J Hematol 2009;90:648-50.

37. Saida S, Yoshida A, Tanaka R, Abe J, Hamahata K, Okumura M, Momoi T. A case of drug-induced hypersensitivity syndrome-like symptoms following HHV-6 encephalopathy. Allergol Int 2010;59:83-6.

38. Conilleau V, Dompmartin A, Verneuil L, Michel M, Leroy D. Hypersensitivity syndrome due to 2 anticonvulsant drugs. Contact Dermatitis 1999;41:141-4.

39. Arevalo-Lorido JC, Carretero-Gomez J, Bureo-Dacal JC, Montero-Leal C, Bureo-Dacal P. Antiepileptic drug hypersensitivity syndrome in a patient treated with valproate. Br J Clin Pharmacol 2003;55:415-6.

40. Roepke S, Treudler R, Anghelescu I, Orfanos CE, Tebbe B. Valproic Acid and hypersensitivity syndrome. Am J Psychiatry 2004;161:579.

41. Augusto JF, Sayegh J, Simon A, Croue A, Chennebault JM, Cousin M, Subra JF. A case of sulphasalazine-induced DRESS syndrome with delayed acute interstitial nephritis. Nephrol Dial Transplant 2009;24:2940-2.

42. Michel F, Navellou JC, Ferraud D, Toussirot E, Wendling D. DRESS syndrome in a patient on sulfasalazine for rheumatoid arthritis. Joint Bone Spine 2005;72:82-5.

43. Tohyama M, Yahata Y, Yasukawa M, Inagi R, Urano Y, Yamanishi K, Hashimoto K. Severe hypersensitivity syndrome due to sulfasalazine associated with reactivation of human herpesvirus 6. Arch Dermatol 1998;134:1113-7.

44. Watanabe H, Tohyama M, Kamijima M, Nakajima T, Yoshida T, Hashimoto K, Iijima M. Occupational trichloroethylene hypersensitivity syndrome with human herpesvirus-6 and cytomegalovirus reactivation. Dermatology 2010;221:17-22.

45. Huang H, Kamijima M, Wang H, Li S, Yoshikawa T, Lai G, Huang Z, Liu H, Chen J, Takeuchi Y, Nakajima T, Li L. Human herpesvirus 6 reactivation in trichloroethylene-exposed workers suffering from generalized skin disorders accompanied by hepatic dysfunction. J Occup Health 2006;48:417-23.

46. Morimoto T, Sato T, Matsuoka A, Sakamoto T, Ohta K, Ando T, Ikushima S, Hagiwara K, Matsuno H, Akiyama O, Oritsu M. Trimethoprim-sulfamethoxazole-induced hypersensitivity syndrome associated with reactivation of human herpesvirus-6. Intern Med 2006;45:101-5.

47. Hubiche T, Milpied B, Cazeau C, Taieb A, Leaute-Labreze C. Association of Immunologically Confirmed Delayed Drug Reaction and Human Herpesvirus 6 Viremia in a Pediatric Case of Drug-Induced Hypersensitivity Syndrome. Dermatology 2011;222:140-141.

48. Mahe E, Bodemer C, Dupic L, Hubert P, Lacaille F, Goulet O, Leruez-Ville M, Fraitag S. Drug-induced hypersensitivity syndrome associated with primary Epstein-Barr virus and human herpesvirus 6 infections in a child intestinal transplant recipient. Transplantation 2004;77:479-80.

49. Tamagawa-Mineoka R, Katoh N, Nara T, Nishimura Y, Yamamoto S, Kishimoto S. DRESS syndrome caused by teicoplanin and vancomycin, associated with reactivation of human herpesvirus-6. Int J Dermatol 2007;46:654-5.

50. Teraki Y, Murota H, Izaki S. Toxic epidermal necrolysis due to zonisamide associated with reactivation of human herpesvirus 6. Arch Dermatol 2008;144:232-5.

51. Fujita Y, Hasegawa M, Nabeshima K, Tomita M, Murakami K, Nakai S, Yamakita T, Matsunaga K. Acute kidney injury caused by zonisamide-induced hypersensitivity syndrome. Intern Med 2010;49:409-13.

52. Kondo K, Kondo T, Okuno T, Takahashi M, Yamanishi K. Latent human herpesvirus 6 infection of human monocytes/macrophages. J Gen Virol 1991;72 ( Pt 6):1401-8.

53. Arbuckle JH, Medveczky MM, Luka J, Hadley SH, Luegmayr A, Ablashi D, Lund TC, Tolar J, De Meirleir K, Montoya JG, Komaroff AL, Ambros PF, Medveczky PG. The latent human herpesvirus-6A genome specifically integrates in telomeres of human chromosomes in vivo and in vitro. Proc Natl Acad Sci U S A 2010;107:5563-8.

54. Daibata M, Taguchi T, Taguchi H, Miyoshi I. Integration of human herpesvirus 6 in a Burkitt's lymphoma cell line. Br J Haematol 1998;102:1307-13.

55. Black JB, Sanderlin KC, Goldsmith CS, Gary HE, Lopez C, Pellett PE. Growth properties of human herpesvirus-6 strain Z29. J Virol Methods 1989;26:133-45.

56. Mardivirin L, Descamps V, Lacroix A, Delebassee S, Ranger-Rogez S. Early effects of drugs responsible for DRESS on HHV-6 replication in vitro. J Clin Virol 2009;46:300-2.

57. Naesens L, Stephens CE, Andrei G, Loregian A, De Bolle L, Snoeck R, Sowell JW, De Clercq E. Antiviral properties of new arylsulfone derivatives with activity against human betaherpesviruses. Antiviral Res 2006;72:60-7.

58. Daigle D, Gradoville L, Tuck D, Schulz V, Wang'ondu R, Ye J, Gorres K, Miller G. Valproic Acid Antagonizes the Capacity of Other HDAC Inhibitors to Activate the Epstein-Barr Virus Lytic Cycle. J Virol 2011.
